# CD44/CD133-Positive Colorectal Cancer Stem Cells are Sensitive to Trifluridine Exposure

**DOI:** 10.1038/s41598-019-50968-6

**Published:** 2019-10-16

**Authors:** Kenta Tsunekuni, Masamitsu Konno, Naotsugu Haraguchi, Jun Koseki, Ayumu Asai, Kazuaki Matsuoka, Takashi Kobunai, Teiji Takechi, Yuichiro Doki, Masaki Mori, Hideshi Ishii

**Affiliations:** 1 Department of Gastrointestinal Surgery, Osaka University Graduate School of Medicine, Osaka, 565-0871 Japan; 2 Department of Medical Data Science, Osaka University Graduate School of Medicine, Osaka, 565-0871 Japan; 3 Present Address: Translational Research Laboratory, Taiho Pharmaceutical Co., Ltd., Tokushima, 771-0194 Japan; 4 Department of Frontier Science for Cancer and Chemotherapy, Osaka University Graduate School of Medicine, Osaka, 565-0871 Japan; 5 Present Address: Department of Surgery and Science, Graduate School of Medical Sciences, Kyushu University, Fukuoka, 812-8582 Japan

**Keywords:** Chemotherapy, Cancer stem cells

## Abstract

Cancer stem cells (CSCs) are involved in metastatic colorectal cancer recurrence, but no effective therapy targeting these cells is currently available. Because trifluridine (FTD)/tipiracil therapy is used for refractory colorectal cancer, we sought to determine whether FTD is effective against CSC-like cells. CD44^+^CD133^+^ high-expressing and other populations of human DLD-1 colon cancer cells were separately isolated through fluorescence-activated cell sorting. The sphere-forming activity of each population and the anti-sphere-forming effects of FTD and fluorouracil (5-FU) on CD44^+^CD133^+^ cells were then measured. CD44^+^CD133^+^ DLD-1 cells formed substantially more spheres than other cells. Moreover, treating CD44^+^CD133^+^ DLD-1 cells with subtoxic concentrations of FTD (1 µM) inhibited sphere formation, and this was superior to the effect of subtoxic concentrations (1 µM) of 5-FU. The associated inhibition rates for FTD and 5-FU were 58.2% and 26.1%, respectively. Further, CD44^+^CD133^+^ DLD-1 cells expressed higher levels of thymidine kinase 1, which is responsible for FTD phosphorylation, than DLD-1 cells, and FTD was incorporated into the DNA of CD44^+^CD133^+^ DLD-1 cells. Thus, our data show that FTD treatment is effective against CSC-like cells and might be applied as CSC-targeting chemotherapy for tumor subtypes with high CD44 and CD133 expression.

## Introduction

Cancer stem cells (CSCs) represent a subpopulation of cells that displays stem cell characteristics and this subset influences tumorigenesis^1^; further, CSCs exhibit diverse cancer-promoting properties such as self-renewal^2^, chemoresistance^3^, and metastatic potential^4,5^. Accordingly, it is considered that CSCs adversely affect patient outcome, but active anti-cancer agents targeting these cells are not currently available in clinical settings.

For the identification of CSCs, one of the first markers of stemness used was the transmembrane glycoprotein CD133, also known as prominin-1^1^^,^^2^, for which expression strongly predicts poorer prognosis; specifically high CD133 levels are inversely correlated with the 5-year overall survival and disease-free survival rates in patients with cancers^6^ including colorectal cancer (CRC). Another putative CSC marker is the cell-surface glycoprotein CD44^7^, which was reported to be an adhesion molecule expressed in cancer stem-like cells^8^. The combined analysis of these putative co-CSC markers, namely CD133 and CD44, was found to improve the discrimination of low- and high-risk cases of CRC, as compared to that with single-marker analyses^9^. Moreover, patients with CD44- and CD133-positive gastric cancer were found to have a poorer survival rate than patients with CD44- and CD133-negative disease^10^.

For CRC, the standard chemotherapy used globally is fluoropyrimidine-, oxaliplatin (L-OHP)-, and irinotecan-based combination regimens with anti-EGFR and anti-VEGF treatment for first-line and second-line settings^11^. In the third- or later-line settings, regorafenib was approved and has been used^12^ in the United States. In addition, The Food and Drug Administration (FDA) has approved pembrolizumab or nivolumab for CRC patients with microsatellite instability-high (MSI-H) tumours and larotrectinib for CRC patients with tumours with neurotrophic tropomyosin receptor kinase (NTRK) fusions in the US. Trifluridine (FTD)/tipiracil (TPI), also known as TAS-102, was also tested for its ability to treat patients with metastatic CRC who were previously treated with chemotherapy based on drugs such as fluoropyrimidine, L-OHP, and irinotecan, among others, and this therapy was found to improve overall survival^13^. After receiving the results of this clinical trial, FTD/TPI was approved and has been used globally in regions such as Japan, the EU, and the US in third- or later-line settings. FTD/TPI is an oral combination drug consisting of FTD, a thymidine analogue, and TPI, a thymidine phosphorylase inhibitor that improves the bioavailability of FTD^14^. Despite the initial success of this agent, its effect on CSC populations was not previously known. Because FTD/TPI is used for refractory CRC, which is considered a CSC-enriched phenotype^15^, we hypothesised that FTD might effectively target CSC-like cells. Here, we investigated whether this drug is effective against CD44- and CD133-highly-expressing (CD44^+^CD133^+^) CRC cells that possess CSC-like properties.

## Results

### Isolation of CD44^+^ CD133^+^, CD44^+^ CD133^−^, CD44^−^ CD133^+^, and CD44^−^ CD133^−^ cells and examination of their sphere-formation ability

**Figure 1:**
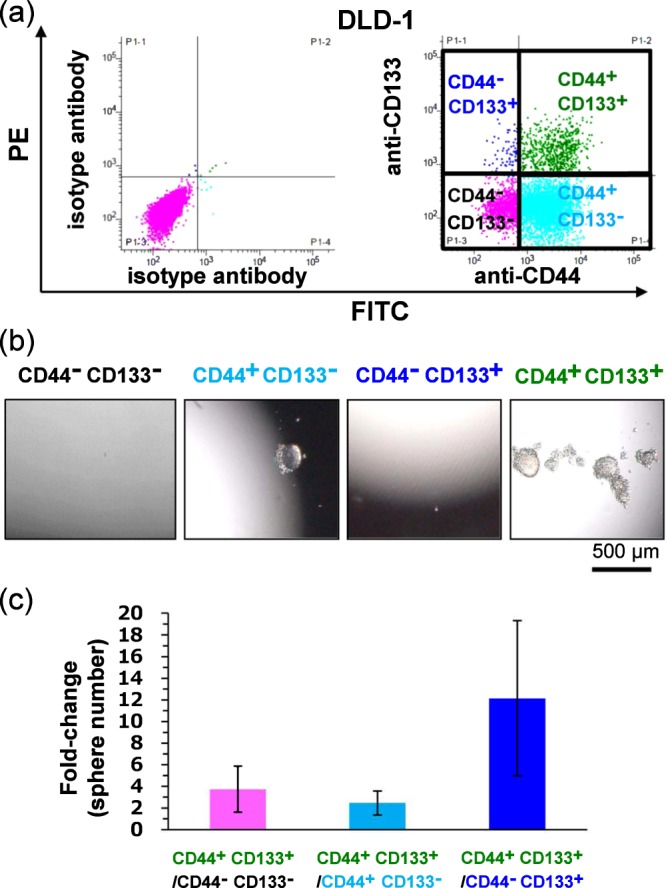
Formation of stem cell spheres after seeding sorted CD44^+^ CD133^+^, CD44^−^ CD133^**−**^, CD44^−^ CD133^+^, and CD44^+^ CD133^**−**^ cells of the colorectal cancer (CRC) DLD-1 cell line. (**a**) Left column shows isotype control and right column shows anti-CD44-FITC and anti-CD133-PE antibody double-staining of DLD-1 cells. The quadrants comprising each population are defined as CD44^**−**^ CD133^+^, CD44^+^ CD133^+^, CD44^−^ CD133^**−**^, and CD44^+^ CD133^**−**^, respectively. (**b**) Representative sphere images of CD44^**−**^ CD133^**−**^ cells, CD44^**−**^ CD133^+^ cells, CD44^+^ D133^**−**^ cells, and CD44^+^ CD133^+^ cells are shown from the left to right column, respectively. (**c**) Sphere numbers determined for CD44^**−**^ CD133^**−**^, CD44^**−**^ CD133^+^, CD44^+^ D133^**−**^, and CD44^+^ CD133^+^ DLD-1 cells. Data points represent means ± SD (n = 6).

To delineate CD44 and CD133 expression in the DLD-1 CRC cell line, we used flow cytometry to measure the expression of these surface molecules after double-staining with anti-CD44-FITC and anti-CD133-PE antibodies. Figure 1a shows DLD-1 flow cytometry data collected after staining and sorting. These cells showed a distribution of CD44 and CD133 expression, with a CD44^+^ CD133^+^ population of 11.3%, a CD44^+^ CD133^−^ population of 65.3%, a CD44^**−**^ CD133^+^ population of 2.1%, and a CD44^−^ CD133^**−**^ population of 21.3%. Cells were isolated using FACS and then transferred into serum-free DMEM/F-12; the isolated CD44^**+**^ CD133^+^, CD44^+^ CD133^**−**^, CD44^**−**^ CD133^+^, and CD44^**−**^ CD133^**−**^ cells were then used for sphere-formation assays to evaluate their stem cell-like properties. Sphere formation in suspension culture is recognised as the prominent characteristic of CSCs and can be used for *in vitro* measurements of CSC function. Here, culturing these cells in serum-free medium in low-adhesion 96-well plates revealed that sphere formation ability was considerably higher in the CD44^+^ CD133^+^ population compared to that in the CD44^**−**^ CD133^**−**^, CD44^+^ CD133^**−**^, and CD44^**−**^ CD133^+^ populations (fold-changes for CD44^+^ CD133^+^ sphere numbers relative to that with other populations were 3.7, 2.5, and 12.1 respectively; Fig. 1c). In addition, sphere sizes were larger in the CD44^+^ CD133^+^ population than in other populations (Fig. 1b). The results indicate that CD44^+^ CD133^+^ populations exhibit the most stem cell-like properties compared to other populations.

### Anti-proliferative effect of FTD on isolated CD44^+^ CD133^+^ cells

**Figure 2:**
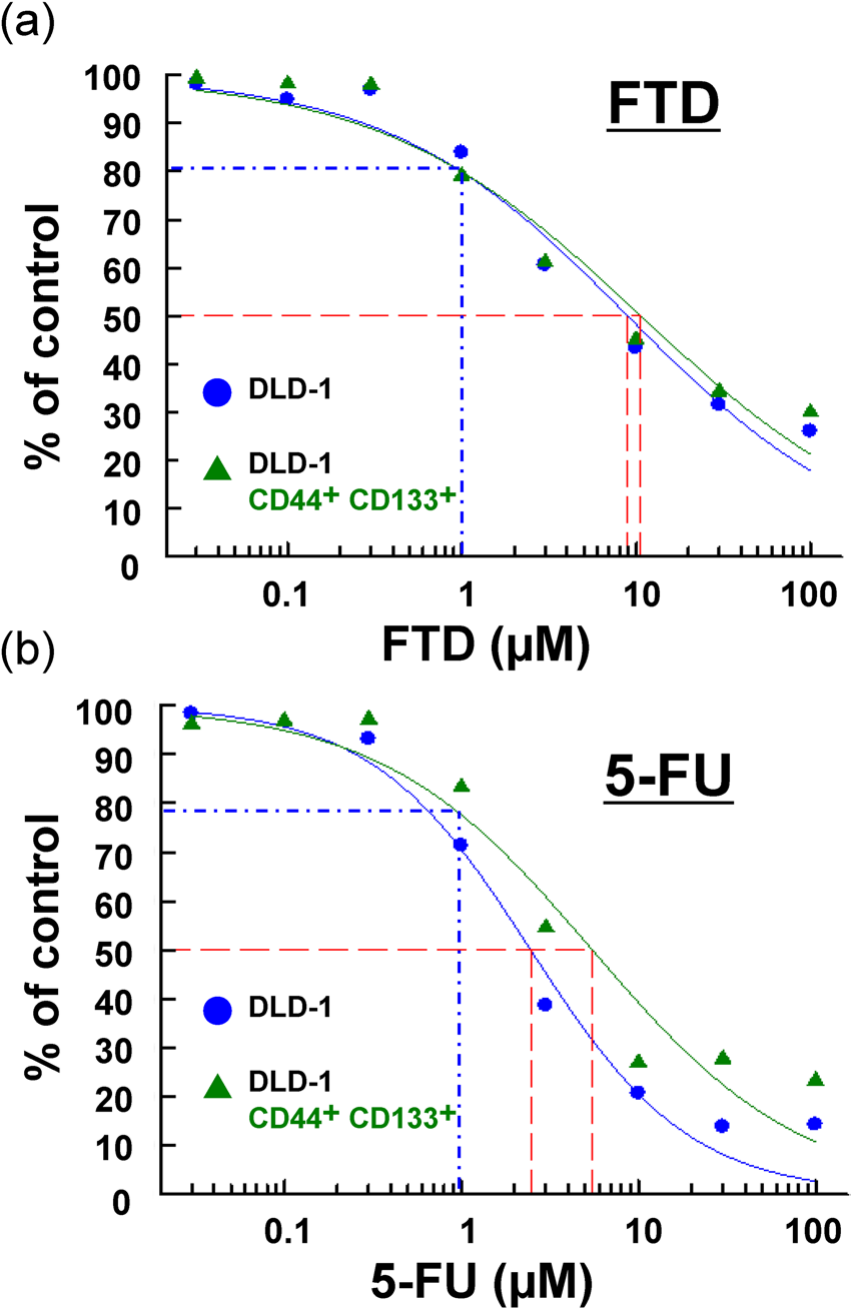
Antiproliferative effect of trifluridine (FTD) on isolated CD44^+^ CD133^+^ cells. Sorted CD44^+^ CD133^+^ cells (shown in Fig. 1) and unsorted DLD-1 cells shown here were cultured with various concentrations of FTD (**a**) and fluorouracil (5-FU) (**b**). Cell viability was determined using crystal violet staining based on at least three independent experiments. Data points represent means ± SD (n = 3). Blue dashed line represents estimated viability determined for 1 µM FTD and 5-FU; values were estimated using a fitting curve in the logistic model. Red dashed line represents estimated IC_50_ values.

We next investigated whether FTD was effective against CSC-like CD44^+^ CD133^+^ DLD-1 cells. The antiproliferative effect of FTD on these cells was investigated by performing cytotoxicity tests with crystal violet staining on CD44^+^ CD133^+^ (depicted in Fig. 1a) and unsorted DLD-1 cells. After 72 h of treatment, FTD was effective against both cell populations, with the calculated IC_50_ values being 10.7 and 8.9 μM, respectively (Fig. 2a). In contrast, resistance toward 5-FU was higher for CD44^+^ CD133^+^ DLD-1 cells (IC_50_ = 5.5 μM) than for unsorted DLD-1 cells (IC_50_ = 2.5 μM); the fold-change in IC_50_ was 2.2 for 5-FU and 1.2 for FTD (Fig. 2b). These results indicate that FTD is effective against a CD44^+^ CD133^+^ CSC-like population.

### FTD treatment and sphere-formation activity

**Figure 3:**
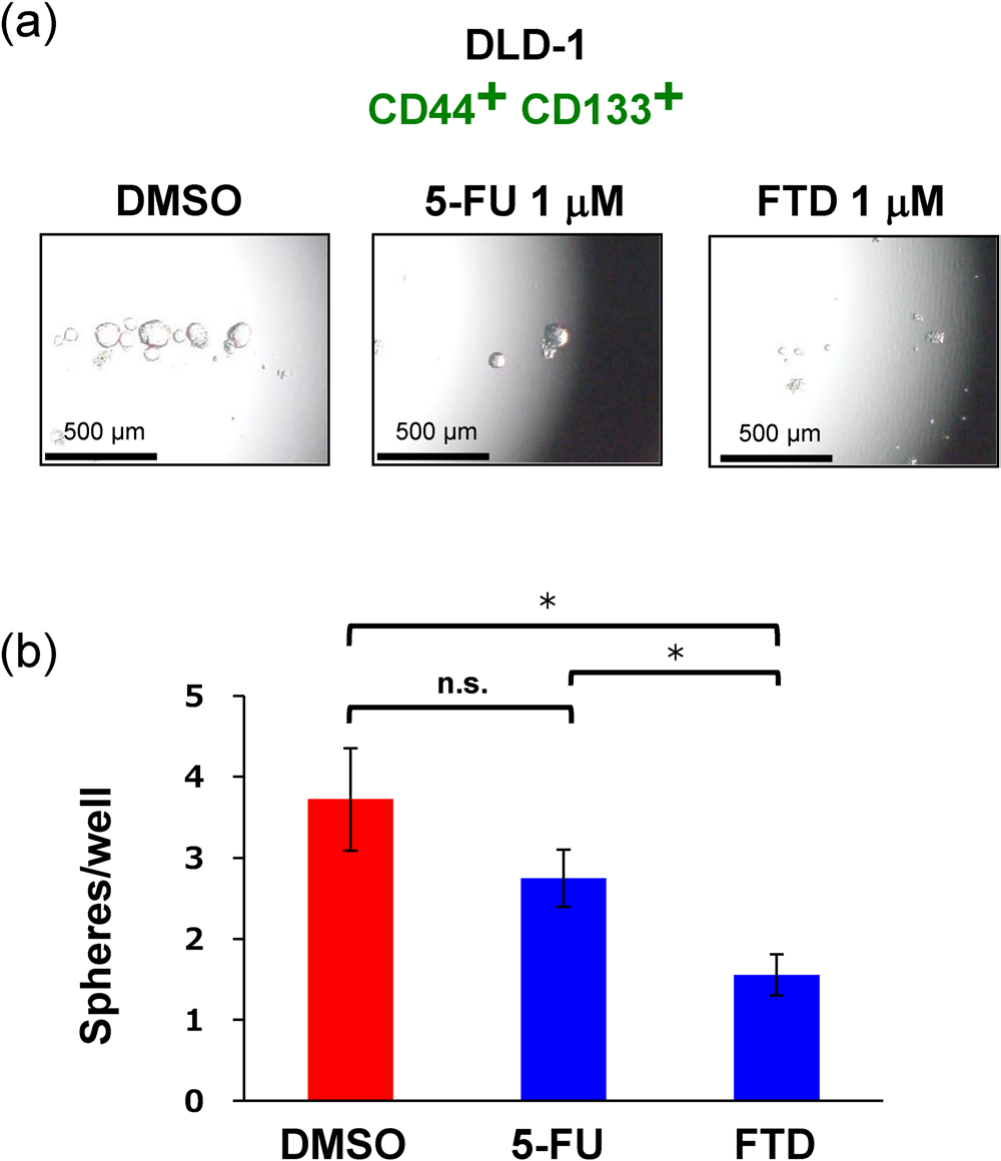
FTD efficacy toward sphere-forming activity of CD44^+^ CD133^+^ cells. Sphere culture was performed using the CD44^+^ CD133^+^ population of the DLD-1 cell line. (**a**) Images of cell spheres treated with DMSO (Control), 1 μM 5-FU, and 1 μM FTD; (**b**) Respective sphere numbers. Data points represent means ± SD (n = 6); Student’s *t*-test, *P < 0.05, n.s.: not significant.

Next, to investigate the effect of FTD treatment on the sphere-forming capacity of CD44^+^ CD133^+^ DLD-1 cells, we performed sphere-formation assays on cells treated with FTD and 5-FU (Fig. 3a,b). As compared to the number of spheres in control samples (DMSO treatment), fewer spheres were present in cells treated with FTD at 1 μM, but not in those treated with 5-FU at 1 μM (Fig. 3b). Both drug concentrations used in this study were sub-toxic (estimated viability determined in the presence of FTD and 5-FU based on cytotoxicity assays with crystal violet staining at 72 h was 79.9% and 77.6%, respectively; both values were estimated based on a fitting curve in the logistic model). Thus, the efficacy of FTD was greater than that of 5-FU in terms of sphere-formation activity, although the cytotoxic effects of both drugs at 1 μM were comparable.

### Thymidine kinase 1 expression in CD44^+^ CD133^+^ cells

**Figure 4:**
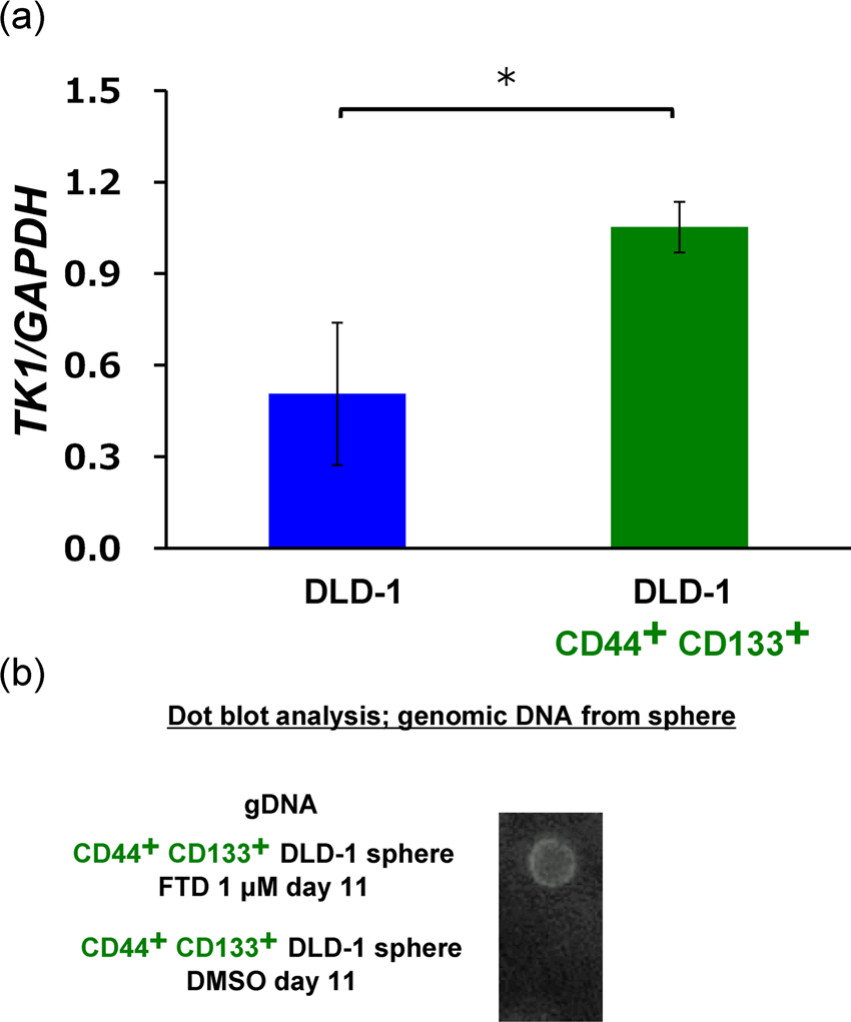
Thymidine kinase (TK1) expression and FTD incorporation in CD44^+^ CD133^+^ DLD-1 cells. (**a**) TK1 expression in CD44^+^ CD133^+^ cells. *TK1* levels in DLD-1 cells and CD44^+^ CD133^+^ DLD-1 cells were assayed using qRT-PCR and normalized against *GAPDH* levels. Data points represent means ± SD of triplicate determinations; Student’s *t*-test, *P < 0.05. (**b**) Dot plot analysis of FTD incorporation into the genomic DNA of CD44^+^ CD133^+^ DLD^-^1 spheres. CD44^+^CD133^+^ DLD-1 cells were cultured in the presence of 1 μM FTD or DMSO for 11 days in sphere-formation assay conditions and genomic DNA was purified. Purified DNA (1 ng) was denatured with an alkaline solution (0.1 N NaOH), spotted onto a Hybond-N^+^ membrane, and blotted with anti-BrdU antibodies.

FTD was previously reported to be phosphorylated by thymidine kinase 1 (TK1) and incorporated into DNA^16^. In addition, FTD incorporation into DNA is the main mechanism underlying its anti-tumour effects^17^. Thus, to further investigate the inhibitive effect of FTD on the sphere-forming capacity of CD44^+^ CD133^+^ DLD-1 cells, we performed real-time PCR analysis to determine the expression of *TK1* using CD44^+^ CD133^+^ DLD-1 cells and unsorted cells. As compared to that in unsorted DLD-1 cells, CD44^+^ CD133^+^ DLD-1 cells exhibited 2.3-fold increased *TK1* expression (Fig. 4a).

### FTD incorporation into the DNA of CD44^+^ CD133^+^ spheres

Finally, because *TK1* was highly expressed in CD44^+^ CD133^+^ DLD-1 cells, we hypothesised that FTD could be incorporated into the DNA of these cells to inhibit their sphere-forming activity. Hence, we investigated the incorporation of FTD into the DNA of CD44^+^ CD133^+^ DLD-1 spheres. Detectable spheres were formed approximately 10 days after cell seeding. Further, after sorting CD44^+^ CD133^+^ DLD-1 cells, the expression of both markers was maintained for 14 days (data not shown). Therefore, the genomic DNA of CD44^+^ CD133^+^ DLD-1 spheres treated with 1 µM FTD was isolated after 11 days, and this compound was detected by dot plot analysis using an FTD-cross-reacting anti-BrdU antibody^18^. Indeed, FTD was detected in the DNA of treated CD44^+^ CD133^+^ DLD-1 spheres but not in the DNA of DMSO (control)-treated cells (Fig. 4b). This result indicates the contribution of FTD DNA-incorporation to its inhibitory effect on sphere-forming activity.

## Discussion

In this study, we showed that CD44^+^ CD133^+^ DLD-1 cells exhibit the highest sphere-forming activity compared to CD44^**−**^ CD133^**−**^, CD44^+^ CD133^**−**^, and CD44^−^ CD133^+^ DLD-1 cells, as reported previously for HCT-116 cells^19^. Here, isolated CD44^+^ CD133^+^ DLD-1 cells also exhibited increased resistance to 5-FU but not FTD as compared to that in unsorted DLD-1 cells based on a sensitivity test (Fig. 2a,b); moreover, FTD but not 5-FU treatment inhibited sphere-forming activity against these cells at a subtoxic concentration (Fig. 3a,b). These results indicated that FTD is effective against CSC-like cells, unlike 5-FU.

**Figure 5:**
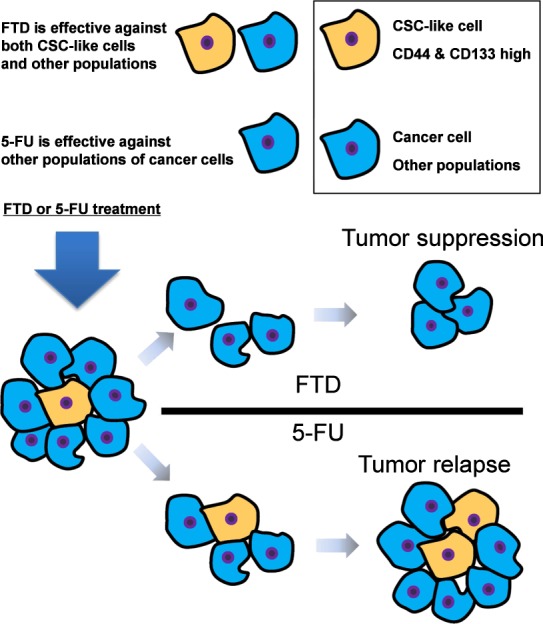
Schematic view of the efficacy of FTD treatment on CD44^+^ CD133^+^ cells. FTD is effective against cancer stem cell (CSC)-like (CD44^+^ CD133^+^) cells, but CSC-like cells show resistance toward 5-FU. FTD could be readily incorporated into the DNA of CSC-like cells. Consequently, FTD might exert an anti-sphere-forming effect on CSC-like cells, unlike 5-FU.

In a previous study, a CSC-like CD44-stably expressing MKN28 gastric cancer cell line was reported to have resistance to cisplatin and docetaxel^8^ and CSC-like CD133-highly expressing primary colon cancer cells were reported to exhibit resistance to 5-FU and L-OHP^3^. Among DLD-1 cells, a CD133-expressing population was also found to be more resistant to 5-FU treatment compared to that with unsorted cells^20^. Further, 5-FU treatment was found to induce an increase in CD44 and CD133 expression, and CD44- and CD133-highly-expressing cells were reported to exhibit stem cell-like properties and resistance to 5-FU^15^. In CSC-like cells, one drug resistance mechanism, with respect to 5-FU and other drugs, was reported to be partially dependent on TRAIL-induced death cell, which was inhibited by the expression of anti-apoptotic proteins^3^. CSC-like CD133^+^ cells are also enriched in anti-apoptotic proteins and are resistant to 5-FU^21^. Our study showed that unlike 5-FU, FTD was effective against CD44^+^CD133^+^ cells. Hence, another mechanism underlying the response of CSC-like cells (such as CD44^+^CD133^+^ cells) to FTD treatment, compared to that associated with 5-FU, is suggested. Poorly-differentiated human hepatocellular carcinoma cells, which present an overlapping stem cell-like phenotype with CSCs, have been reported to show the upregulated expression of pyrimidine metabolism-associated rate-limiting enzymes including TK1^22^. In addition, CD44 expression is positively correlated with TK1 expression in triple negative breast cancer^23^. Moreover, *TK1* mRNA was found to be upregulated in CD133-expressing human embryonic stem cells^24^. Because TK1 catalyses FTD phosphorylation^16^, it might be readily incorporated into the DNA in TK1-upregulated CSC-like populations. In this study, *TK1* mRNA expression was higher in CD44^+^ CD133^+^ DLD-1 cells than in other populations (Fig. 4a), and indeed, FTD was incorporated into the DNA of CD44^+^ CD133^+^ DLD-1 spheres (Fig. 4b). FTD incorporation into the DNA of CSC-like cells might explain the anti-sphere forming activity of FTD toward 5-FU-resistant CSC-like spheres. Although CSC-like cells could be enriched in anti-apoptotic proteins, incorporation of the nucleotide analogue FTD into the DNA might provoke the interruption of DNA replication and delay the self-renewal of CSC-like cells. As a consequence, a preferential antiproliferative effect might be sustained in CSC-like cells (Fig. 5).

There are several limitations to this study. First, we used only DLD-1 cells, which were associated with the highest sorting efficacy for both CD44^**−**^ CD133^**−**^ and CD44^+^ CD133^+^ populations among three cell lines tested, also including HCT-116 and RKO cells (data not shown), to determine the efficacy of FTD toward CD44^+^ CD133^+^ CRC cells. Moreover, our study showed that FTD exerted anti-sphere-forming activity toward CSC-like CD44^+^ CD133^+^ cells. However, these results are based only on *in vitro* experiments and therefore must be confirmed using *in vivo* assays and clinical studies. Accordingly, to investigate FTD efficacy in CSC-like CD44^+^ CD133^+^ cells, we are planning an *in vivo* serial transplantation study to evaluate whether it can decrease tumorigenic capacity.

Despite the limitations, our study indicated for the first time that unlike other anti-cancer drugs, FTD might be effective against CSC-like cells expressing high levels of CD44 and CD133. These effects on CSC-like cells might explain the observed improvement in overall survival in patients treated with FTD/TPI in the clinical setting^13^. For gastric cancer, FTD/TPI has been used to treat patients with heavily-pre-treated metastatic gastric/gastroesophageal junction (GEJ) cancers who have progressed or are intolerant to previous lines of therapy, and this was found to improve overall survival^25^. In gastric cancer, CD44 and CD133 were also reported to be relevant to chemoresistance^26^. Therefore, similarly, the improvement in overall survival could be due to the efficacy of FTD against CSC-like cells exhibiting high CD44 and CD133 expression and might be applicable for GEJ, as well as CRC. In addition, a similar response rate between FTD/TPI + bevacizumab and capecitabine + bevacizumab was confirmed in the TASCO-1 clinical trial conducted based on a first-line setting^27^; thus, the possibility that this drug might also be useful in an adjuvant setting (stem-like cell killing effect) should also be considered. Furthermore, this study suggests a potential role for FTD/TPI in chemo-preventive therapy due to its efficacy against drug-resistant (or at least 5-FU-resistant) CD44^+^ CD133^+^ CSC-like cells at subtoxic, low doses (see Figs 2 and 3). Although further investigation is required to clarify the efficacy of this drug for tumors exhibiting CSC-like properties in the clinical setting, our study indicates that FTD-based treatment might lead to improved clinical outcomes for patients with tumours exhibiting high CD44 and CD133 expression.

## Materials and Methods

### Cell lines and culture conditions

The DLD-1 human colon cancer cell line was obtained from the American Type Culture Collection and grown in Dulbecco’s modified Eagle’s medium (DMEM; Sigma-Aldrich, Tokyo, Japan) supplemented with 10% fetal bovine serum (Life Technologies, Carlsbad, CA, USA), 100 U/mL penicillin, and 100 µg /mL streptomycin (Life Technologies). Cells were grown at 37 °C in a humidified atmosphere with 5% CO_2_. Cell lines were maintained as mentioned, and all lines were verified based on short tandem repeats before the study; all experimental procedures were performed using exponentially growing cells.

### Chemicals

FTD, 5-FU were purchased from Tokyo Chemical Industry (Tokyo, Japan).

### Cytotoxicity assay

Cell lines were seeded at a density of 2000 cells/well in 96-well plates, precultured for 24 h, and then exposed to FTD (0.03, 0.1, 0.3. 1, 3, 10, 30, 100, 300 μM) or 5-FU (0.03, 0.1, 0.3. 1, 3, 10, 30, 100, 300 μM) for 72 h. The *in vitro* cytotoxic effects of FTD and 5-FU were evaluated based on crystal violet staining. For this, cells were fixed with 2% glutaraldehyde for 20 min, stained with 0.05% crystal violet (Wako, Tokyo, Japan) in 20% methanol for 20 min, and rinsed with tap water; subsequently, the plates were dried on paper for 1 h and then 100 μL of a 1:1 mixture of ethanol and 0.1 M sodium dihydrogen phosphate was added to each well. Absorbance was measured at 595 nm by using an EnSpire system (PerkinElmer, Waltham, MA, USA). The FTD and 5-FU concentration that inhibited cell growth by 50% (IC_50_) was calculated from the generated regression lines.

### Fluorescence-activated cell sorting (FACS) analysis of cells

To analyse and sort based on the expression of CD44 and CD133, cells were routinely cultured, harvested, digested with Accutase (Life Technologies), and resuspended in staining buffer (1 × 10^7^ cells in 1 mL), and then incubated with antibodies (mouse anti-human CD44-FITC (fluorescein isothiocyanate) and mouse anti-human CD133-PE (Phycoerythrin); Miltenyi Biotec, Bergisch Gladbach, NW, Germany) for 10 min according to the manufacturer’s procedures. After staining, cells were analysed and sorted by flow cytometry using a BD FACSARIA cell sorter and a BD FACSMelody Cell Sorter (Becton Dickinson, Franklin Lakes, NJ, USA).

### Sphere-formation assay

Cells were seeded into 96-well flat-bottom ultra-low-attachment culture dishes (Corning, Corning, NY, USA) at 100–500 cells/well in DMEM/F-12 serum-free medium (Life Technologies) containing 20 ng/mL h-EGF, 20 ng/mL h-bFGF (Sigma-Aldrich), 2% B27 Supplement, and 1% N-2 Supplement (Life Technologies) with or without FTD and 5-FU. After 14 to 40 days, the size of spheroid colonies was measured under a microscope, and colonies with a diameter exceeding 50 μm were counted.

### Real-time quantitative reverse transcription-polymerase chain reaction (qPCR)

To assay mRNA expression, cDNA was synthesized from extracted total RNA, and then qPCR was performed using the synthesized cDNA products, ReverTra Ace qPCR RT Master Mix (TOYOBO, Tokyo, Japan), and the SYBR qRT-PCR Kit (Clontech, Mountain View, CA); cDNA was amplified using primers specific for *TK1* (forward: 5ʹ-AGAGTACTCGGGTTCGTGAACTT-3ʹ; reverse: 5ʹ-CACTTGTACTGAGCAATC-3ʹ). *GAPDH* was used as a normalisation control (primers, forward: 5ʹ-GAGTCAACGGATTTGGTCGT-3ʹ; reverse: 5ʹ-TTGATTTTGGAGGGATCTCG-3ʹ). Relative expression was calculated using the CT-based calibrated standard-curve method. The calculated values were then normalised against *GAPDH* expression.

### Dot plot analysis

Genomic DNA was purified from CD44^+^ CD133^+^DLD-1 spheres cultured in the presence of 1 μM FTD for 11 days using the NucleoSpin® Tissue kit (MACHEREY-NAGEL, Düren, NN, Germany). Equal amounts (1 ng: DLD-1 in 2 μL) of denatured (0.1 N NaOH for 5 min at room temperature (RT)) genomic DNA were spotted onto a Hybond-N^+^ blotting membrane (GE Healthcare Life Sciences, Little Chalfont, Chicago, IL, USA). The membranes were baked at 80 °C for 2 h, blocked with Blocking-One solution (Nacalai Tesque, Kyoto, Japan), and blotted with diluted anti-BrdU antibody 3D4 (Cat#555627) from BD Biosciences (San Jose, CA, USA). Chemiluminescent signals were captured using the ChemiDoc™ Touch system (BIO-RAD, Hercules, CA, USA).

### Statistical analysis

Statistically significant differences were identified using the Student’s *t*-test as appropriate. All statistical analyses were performed using JMP Pro 9 software (SAS Institute, Cary, NC, USA).

## Data Availability

The datasets generated during and/or analysed during the current study are available from the corresponding author upon reasonable request.
